# Tuberculosis1Read before the meeting of the Illinois Veterinary Medical and Surgical Association, August, 1899.

**Published:** 1899-09

**Authors:** V. G. Hunt

**Affiliations:** Arcola, Ill.


### TUBERCULOSIS.[1]

By V. G. Hunt, V.S.,
ARCOLA, ILL.

By request I present this paper to the Illinois Veterinary Medical and Surgical Association, not that I have solved any of the unsolved problems or have any pet theories, or to defend any old and popular theories that have only the sanction of antiquity to recommend them, for simply after forty years of active practice among the flocks and herds of central Illinois, I merely give my own convictions. And would it not, indeed, be strange if I did not have some theories indelibly stamped in my mind, however erroneous they may be? If we are to credit our daily papers, tuberculosis is alarmingly on the increase. The livestock interests demand the most earnest consideration of the professional men. Men who have all the scholastic attainments of the age, and have grown gray

[1] Read before the meeting of the Illinois Veterinary Medical and Surgical Association, August, 1899.

in active practice, give us but little hope as to treatment, but stand powerless spectators, recommending the slaughter of the whole herd where there is an outbreak. Let us hope within the next decade some remedy may be found that will stay its ravages. Let investigation be the watchword, and future generations will rise up and call us blessed. Without doubt every drug to be found in therapeutic arsenals has been tried and will continue to be tried, notwithstanding the unsatisfactory results. Faith plays no part in our profession.

As to the contagiousness of tuberculosis some curious facts confront us. Some large dairies furnish milk for the cities where the principal diet of small children is milk. The slightest suspicion of tuberculosis is kindled to a flame of discovery. The whole herd is immediately slaughtered, and the autopsy confirms the diagnosis of tuberculosis. But nowhere upon investigation can any immediate casuality be found. It is a well authenticated fact that in many places in Europe the poor class seldom taste meat, and tuberculosis does exist there. Avaricious owners dispose of tuberculous animals to the butcher, the diseased part being carefully dissected out. There have been no casualties reported, and some veterinarians of worldwide reputation sanction its use. However revolting this would be to the average American, it is practised in Europe. Cannot we say that this disease is practically hereditary, or certain conformations are more susceptible to tnberculosis. The higher the animal is bred the more liable it is to contract this disease. But just how it enters the body we are not clear. Conflicting theories have made us somewhat skeptical. We find this disease often in the lungs, the liver, the bowels, the lymphatic glands, and the spleen. In dissection no very extensive lesions are found. In some localities we find these tumors in a capsule, and for the time being are perfectly inert until some exciting cause arouses them to action, and they run their course with fearful rapidity.

The livestock interest is too great to be trifled with. To stamp out this disease men should be appointed who have extensive judgment as well as education, with practical experience. Here the scientific scholar, and one whom dame Nature has been most liberal with brains, never minding his ability as a political worker or ward heeler. Purity in politics at this time, of all others, is demanded. Sensational articles not supported by facts should be withheld. Our Livestock Commission have a herculean task before them, and from what we can learn are conscientious gentlemen. May the profession hold up their hands as Moses did that Israel might prevail. Some writers throw out the hint that tuberculosis, like some disease in man, that passing through coming generations, may become so modified that it will seldom crop out, and then in its mildest form. Be this as it may, it will be a legacy handed down that will hardly be appreciated. Let every veterinarian in this whole land do some original thinking. Never throw cold water on honest effort. Charity is a divine attribute. Let us cultivate this virtue. However humble our station in life may be, we have a certain mission to perform. Just how great is the danger of contagion is somewhat in doubt—largely speculative. So also to discriminate just when the disease renders the animal unfit for human food requires the nicest discrimination. The "Greenhut Trial," a short time since in Peoria, where a large number of cattle were destroyed with actinomycosis, or lumpy-jaw, is fresh in the memory of the profession. The expert testimony shocked the sensibilities of every lover of justice. The views of the profession have been somewhat modified since that illustrious trial. Is it not possible that our radical views of tuberculosis may undergo a similar change? Some of our theories, like our theology, largely speculative, can neither be affirmed nor denied. Largely a creature of faith, while some build up others tear down, but in time slow reason triumphs. Truth is oftener stranger than fiction.

---

The Board of Agriculture of Great Britain has formulated the following regulations as to the admission of dogs from Ireland: Prohibits the landing of unlicensed dogs; imposes a quarantine of six months, unless after ninety days' detention a verterinary certificate is produced to show that the animal is in good health and may safely be removed inland.

Waldo F. Brown, in the *Country Gentleman*, says: "From a stylish mare of good breeding I have raised two mule colts, after which I again bred the mare to a fine carriage-horse. The filly is now past two years old, and is as handsome a colt as I ever raised, showing no 'mulish appearance' whatever." This is contrary to the views of some writers and authorities on the subject.

Kansas has quarantined 250 Texas cattle on the alleged discovery of Texas fever.

